# Landscape-Scale Analysis of Wetland Sediment Deposition from Four Tropical Cyclone Events

**DOI:** 10.1371/journal.pone.0050528

**Published:** 2012-11-21

**Authors:** Andrew W. Tweel, R. Eugene Turner

**Affiliations:** Department of Oceanography and Coastal Sciences, School of the Coast and Environment, Louisiana State University, Baton Rouge, Louisiana, United States of America; Kenya Medical Research Institute - Wellcome Trust Research Programme, Kenya

## Abstract

Hurricanes Katrina, Rita, Gustav, and Ike deposited large quantities of sediment on coastal wetlands after making landfall in the northern Gulf of Mexico. We sampled sediments deposited on the wetland surface throughout the entire Louisiana and Texas depositional surfaces of Hurricanes Katrina, Rita, Gustav, and the Louisiana portion of Hurricane Ike. We used spatial interpolation to model the total amount and spatial distribution of inorganic sediment deposition from each storm. The sediment deposition on coastal wetlands was an estimated 68, 48, and 21 million metric tons from Hurricanes Katrina, Rita, and Gustav, respectively. The spatial distribution decreased in a similar manner with distance from the coast for all hurricanes, but the relationship with distance from the storm track was more variable between events. The southeast-facing Breton Sound estuary had significant storm-derived sediment deposition west of the storm track, whereas sediment deposition along the south-facing coastline occurred primarily east of the storm track. Sediment organic content, bulk density, and grain size also decreased significantly with distance from the coast, but were also more variable with respect to distance from the track. On average, eighty percent of the mineral deposition occurred within 20 km from the coast, and 58% was within 50 km of the track. These results highlight an important link between tropical cyclone events and coastal wetland sedimentation, and are useful in identifying a more complete sediment budget for coastal wetland soils.

## Introduction

Recent hurricanes caused significant damage to coastal communities, and brought increased attention to the role of coastal wetlands in buffering storm surge [1], [2]. This buffering capacity is due to the reduction in wave energy as the incoming storm surge moves across wetlands and shallow coastal waters [3], [4]. Sediments are transported across the coastal landscape as the potential for sediments to become suspended rises with storm energy and declines as wave energy is reduced. The amount of re-distributed sediment can be huge - up to 10^8^ t (metric tons) deposited across hundreds of km^2^ of wetlands – and dense [5]. Up to 68 g cm^−2^ of sediment, for example, were deposited on Louisiana's coastal wetlands during Hurricane Katrina [5].

**Figure 1:**
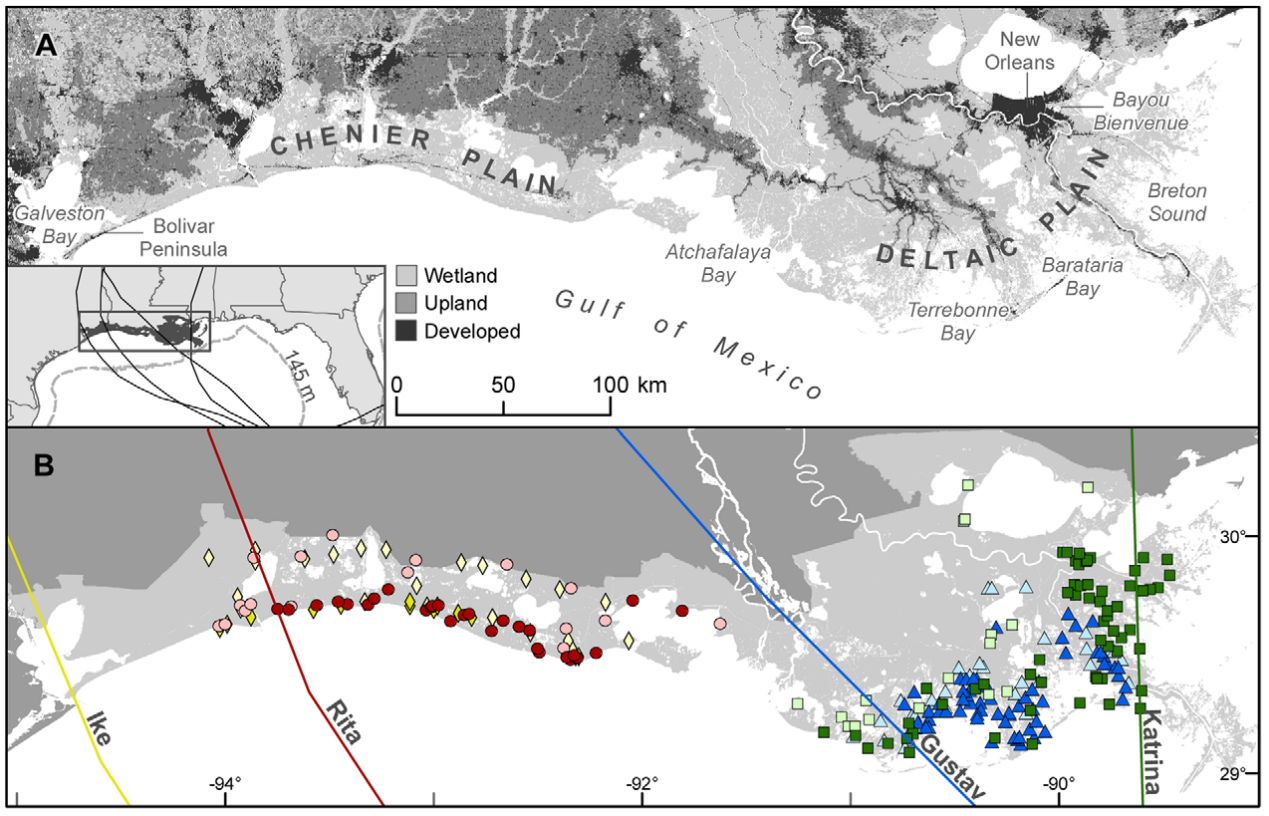
Study area, place names discussed in text, and field sampling locations. A. Map showing locations of geographic names used in text. Inset shows general location of study area, hurricane paths (black lines), and 145 m isobath (dotted line). B. Sampling locations and storm paths for Hurricanes Katrina (green square), Rita (red circle), Gustav (blue triangle), and Ike (yellow diamond). The dark colored symbols mark observed deposition and light colored symbols mark observation of no sediment. Sampling for Hurricane Ike did not include Texas, although considerable deposition occurred [21].

These newly-deposited sediments may have been transported from as far offshore as the continental slope. The infrequent, but intense, storm surge events regularly punctuating the microtidal Louisiana coastal zone can reach heights exceeding normal tidal cycles by several orders of magnitude. Data from offshore buoys during Hurricane Katrina, for example, show that the maximum wave height 100 km east of the hurricane path was about 17 meters, with a wave period exceeding 14 seconds [6]. The shear stress produced by such waves is capable of suspending grains at least as large as coarse sand (1 mm) at water depths greater than 140 m [7], [8]. The sea floor depth in this area is also around 140 meters, which is the approximate depth where the continental shelf transitions to deeper water along the Gulf Coast (Fig. 1). Therefore, Louisiana continental shelf sediments, much of which once flowed down the Mississippi River, may be available for transport by waves associated with tropical cyclone events.

The suspended sediment is carried in waves whose fate depends on the bathymetry encountered as they propagate shoreward. Massive hurricane depositional events have been observed offshore of coastal Louisiana. Following hurricanes Katrina and Rita, the deposition on the inner continental shelf was estimated to be 1160 MMT (million metric tons) - 10 times the average annual deposition rate for that area [9]. Similar depositional events were observed following Hurricane Lili in 2002 [10], and Hurricane Ivan in 2004 [11]. The characteristics of these newly deposited sediments did not appear to be from inshore sources such as wetlands or ponds [9]. East of the Mississippi River, at depths of 4 to 10 meters, sediment cores revealed the preservation of event layers that corresponded to large storm events up to 50 years prior [12].

Sediment deposition in inshore waters has also been reported following hurricane events. Sediment accumulations up to 2 m thick were found in tidal creeks of the Florida Everglades following Hurricane Andrew in 1992 [13]. The storm surge associated with Hurricane Andrew's Louisiana landfall also deposited several centimeters of mud in coastal Louisiana marsh ponds located 5 km northwest of Terrebonne Bay and 45 km east of the storm path [14]. More recently, sediment deposition of up to 10 cm was observed in Sister Lake, located 5 km inland from the Gulf of Mexico, following Hurricanes Rita (200 km to the west of Sister Lake) and Katrina (130 km to the east) [15]. These sediments contained up to 42% sand, and were considered to be indicative of high-energy transport and deposition. The results of isotopic analysis of these sediments revealed high ^234^Th activity, which was interpreted to indicate an offshore sediment origin [15].

Sediment deposition on the wetland platform has been more widely studied than subaqueous deposition, possibly because it is more readily distinguished from the existing soil surface. Early reports noted storm surge deposition on wetlands as far back as Hurricane Audrey in 1957 [16], and later studies analyzed sediment deposition following Hurricane Andrew [17], [18]. While mostly observed in Louisiana due to its broad coastal wetland landscape, wetland sedimentation has also been described in Florida after Hurricanes Andrew in 1992 [13], Irene in 1999 [19], and Wilma in 2005 [20]. Wetland sediment deposition was also observed from Texas to the Mississippi and Alabama coasts following the 2005 hurricane season [21], [22].

The deposition of these sediments is influenced by a variety of factors related to various physical and biological conditions, but there is little understanding about the spatial distribution of the sediment deposited, and how it varies from storm to storm. The focus of this analysis is to investigate the spatial distribution of sediments on coastal wetlands following hurricanes at an event or coast-wide scale. We conducted a large-scale spatial analysis, and incorporated an analysis of smaller scale variations by examining the residuals within a landscape-scale model. The objective of this research is to determine how the quality and quantity of sediments deposited during 4 hurricanes in the last 10 years varies spatially within a Louisiana coastal landscape.

## Materials and Methods

We sampled sediments deposited within four months of landfall for Hurricanes Katrina (2005), Rita (2005), Gustav (2008) and Ike (2008) (Fig. 1). These data were compiled into a spatial database that was used to estimate where and how much sediment was deposited in each event.

### Ethics statement

No specific permits were required for the described field studies. We did not sample in any areas marked as private property or that displayed “posted” or “no trespassing” signs.

### Field sampling and laboratory analysis

The field sampling was designed to encompass the entire depositional area following Hurricanes Katrina, Rita, Gustav, and Ike. The entire Louisiana depositional areas were sampled (Fig. 1), but the depositional area of Hurricane Ike was not completely sampled because of the logistical impediments to accessing wash-over areas from the Texas border to Bolivar Peninsula. We distinguished sedimentation from storms that occurred the same season by identifying gaps between the sampled areas where one event layer tapered to no deposition before the other began (Fig. 1). We collected samples by accessing the sites by outboard boat, car, helicopter or airboat at increasing distance from the coast and storm path with the objective of enclosing the sample area with observations of zero deposition on all sides. Sampling was done as quickly as possible following the events, but was not completed until as late as four months after landfall because of the time required to sample such a large area and storm damage to roads and social/economic infrastructure.

Each observation consisted of measuring sediment thickness, percent mineral content, and bulk density. In order to accurately quantify sediment deposition, several preliminary samples were taken before the final sample was collected using the method discussed by Turner and others [5]. The depth of deposition was measured using a ruler. Sediment was collected from the vegetated areas only, and away from the wetland edge, using a modified syringe to take a small core, and therefore a known volume, from which bulk density could be determined. Hurricane-deposited sediment was clearly distinguishable from pre-existing sediment where green blades of marsh grasses were preserved below the new layer, thus indicating very recent deposition. Deposition less than 0.5 cm was difficult to accurately separate from existing detritus, and was considered zero for this analysis. The depositional areas and total amount deposited are, therefore, conservative estimates.

### Sediment characteristics

Sediment samples were dried and analyzed for inorganic content by loss on ignition. Mineral accretion was used to model sediment deposition, and derived by multiplying deposition depth by sample bulk density and percent mineral content to yield mineral accretion in g cm^−2^. Bulk density and mineral content were compared at increasing distances from storm path and the coastline.

We used a subsample from 24 locations impacted by Hurricane Gustav to investigate grain size distributions within the event. The subsample was drawn from an area 30 km east of the track at the head of Terrebonne Bay, where sampling density was greatest, and included a gradient of inorganic sediment from 6.6 g cm^−2^ near the bay to 0.6 g cm^−2^ 19 km inland. Samples were prepared for analysis using standard procedures [23]. Rehydrated sediment was passed through a 250 µm sieve to remove large particles, if any. Organic material was assumed to be hydrodynamically equivalent, and was oxidized with a solution of 30% H_2_O_2_. The sediment samples were then dispersed with 0.05% sodium hexametaphosphate. Sediment grain size distributions were determined using a laser diffraction particle size analyzer (Beckmann Coulter LS 13 320).

### Spatial analysis

All spatial analyses were conducted using ArcInfo 10.0 (ESRI, Redlands, CA, USA). Two distinct interpolation methods were tested to estimate depositional patterns on a coastwide scale. A kriging analysis was not a suitable method when observed deposition lacked significant spatial autocorrelation, as was the case in several locations within the sampling areas. Inverse distance weighting (IDW), which does not assume input data are spatially autocorrelated, was applied to these datasets and provided the most statistically viable results. Inverse distance weighting is considered an exact interpolator, in that the output surface passes through each observation. The areas between sampled locations are estimated based on nearby samples, and are weighted to favor closer samples over more distant observations. We selected model parameters for neighborhood and power that minimized interpolation error. We could only estimate the Louisiana portion of the deposition during Hurricane Ike because the depositional area in Texas was not sampled.

The method used to bound the depositional area was imperfect because the observed areas with zero deposition did not always completely encircle the areas with deposition. Based on decay relationships within areas that were more densely sampled, we applied a 20 kilometer buffer to each sample area, and assumed that no deposition occurred beyond this boundary. This served as the outer bound of spatial interpolation. To analyze Hurricanes Katrina and Gustav we divided the study areas east and west of the Mississippi River, and recombined them following interpolation. All data were processed in North American Datum 1983 Universal Transverse Mercator zone 15 north and a pixel size of 1 km^2^. About 15% of the study area is in Universal Transverse Mercator zone 16-north, but the difference in area computed in its native zone compared to zone 15-north at this latitude was 0.1% in one area tested.

We used United States Fish and Wildlife Service National Wetlands Inventory data to calculate wetland areas because it is consistent in coverage and source data throughout the study area. The mean pixel value (mineral sediment deposition per cm^2^) for each event was multiplied by the area of wetlands in each area to produce an estimate of total deposition. These estimates were formulated for all Louisiana coastal wetlands, and all Texas coastal wetlands to Galveston Bay. The root mean square error, also in units of mineral sediment deposition per cm^2^, from each interpolation was used to calculate interpolation error terms for each estimate.

To estimate the total depositional area from Hurricane Ike, we combined our estimate with that of a previously published estimate that covered the area we were unable to reach [21]. We subtracted the overlapping areas from our model, and then combined the two figures to estimate the total wetland deposition following Hurricane Ike.

### Spatial distribution

In addition to the total sediment deposited per event, we also analyzed the spatial distribution of sediment within each depositional area. We measured the amount of total deposition that occurred within 10 km increments from the storm track and also from open water. Based on preliminary observations that sediment distribution was highest near the open water of the Gulf of Mexico and also large inshore water bodies, we defined the open water buffer as beginning 5 km from land along inshore areas (e.g. Breton Sound, Barataria Bay) and directly along marshes that extend to the Gulf. Land-to-water ratios within each area were also calculated to test if the results reflected differing wetland coverage rather than deposition.

## Results

### Total deposition

**Figure 2:**
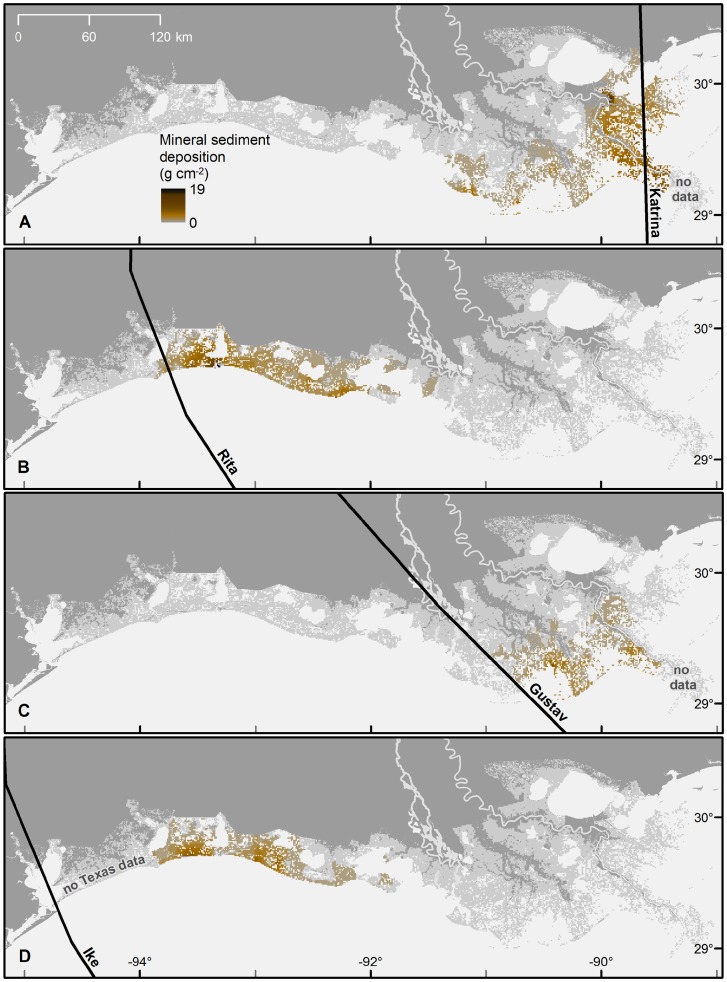
Wetland sediment deposition following four recent hurricanes. Mineral sediment deposition (g cm^−2^) from Hurricanes Katrina (A), Rita (B), Gustav (C), and the Louisiana portion of Ike (D) interpolated using inverse distance weighting at 1 km^2^ resolution.

**Figure 3:**
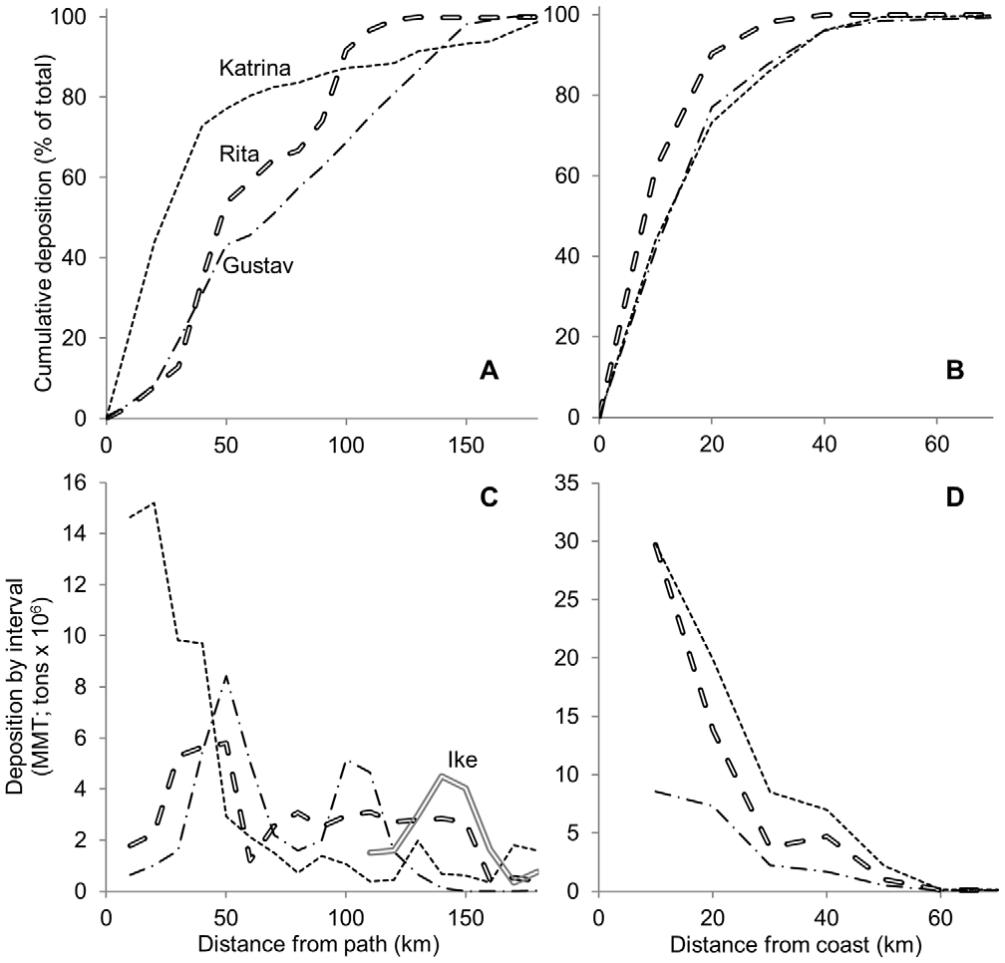
Distribution of mineral sediment with respect to distance from storm path and distance from coastline. The top panels (A, B) show cumulative deposition and bottom panels (C, D) show deposition within each 10 km interval, plotted at the midpoint. The panels at left (A, C) show distance from path and the panels at right (B, D) show distance from coast. Deposition is based on spatially interpolated sediment distribution measured at 10 km increments. Deposition is measured east of the storm path for Gustav, Rita, and Ike, and on both sides for Katrina because of the distinct distribution of sediment. The data for Hurricane Ike in panel C is for the Louisiana portion of the deposition footprint; Texas is excluded.

The storm surges of Hurricanes Katrina, Rita, and Gustav deposited 67.8±8.6, 48.2±8.3, and 20.6±3.9 (estimate ± root mean square error) million metric tons (MMT) of inorganic sediment on coastal wetlands, respectively, when calculated using the IDW method (Fig. 2, Table 1). Hurricane Ike deposited an estimated 32.8±10.9 MMT on Louisiana wetlands alone, but the sampling density was less than the other events and resulted in a greater percent error. The results from the kriging analysis were within 2% of the results from the IDW model for Katrina and Gustav, and 6% for Rita, but some sample areas did not meet the model assumption of spatial autocorrelation. The use of the IDW method tended to reduce data variability, and observations of high deposition were tempered because the final estimate pixel values represent the mean of the observation as well as estimated values possibly driven down by neighboring points. For this reason, the total deposition estimates may be conservative figures because half of the total deposition is estimated to occur in these high deposition areas (Fig. 3).

**Table 1:** Mineral sediment deposition estimates and model results for Hurricanes Katrina, Rita, Gustav, and Ike.

		Wetland deposition			IDW statistics^2^			Most distant observed sedimentation			
	Gulf Coast landfall	Total (MMT)		Model error (MMT)	RMSE^3^ (g cm^−2^)	Mean error (g cm^−2^)	*n*	(km from track)	(km from coast)	Maximum accretion (g cm^−2^)	Percent land in depositional area
Katrina	8/29/2005	67.8	±	8.6	2.4	0.09	77	148	43	20.81	52.19
Rita	9/24/2005	48.2	±	8.3	3.25	0.11	45	166	12	19.95	64.29
Gustav	9/1/2008	20.6	±	3.9	1.19	0.04	110	100	40	7.46	55.40
Ike^1^	9/13/2008	32.8	±	10.9	1.85	0.17	37	214	7	16.73	65.54
Total		169.4	±	31.7							

1 Texas depositional area not sampled.

2 RMSE: root mean square error.

3 IDW: inverse distance weighting, the method used to estimate total deposition.

### Spatial distribution

The storm surge from Hurricane Ike resulted in sedimentation farthest from the storm path (214 km) when compared to the other three storms (Table 1). Sedimentation from Hurricane Katrina reached the farthest inland, however. The observed inland limit of sedimentation was also greater in the two Deltaic Plain events than those in the Chenier Plain. The percent water within the study areas was similar, but Chenier Plain study areas contained less open water (Table 1).

Hurricane Katrina deposited 80% of the total amount within 60 km of the hurricane track, which is more than from Hurricanes Rita or Gustav, (Fig. 3A). By comparison, Hurricanes Rita and Gustav deposited 46% and 59%, respectively, within the same distance. All three of these events deposited over 90% of the total mineral sediment within 140 km of the path. In contrast to Hurricane Katrina, Hurricanes Rita and Gustav deposited the most sediment between 20 and 50 km from the path, with less sediment being deposited closer to the path (Fig. 3B). The peak sedimentation from Hurricane Katrina occurred nearest the track.

The patterns of deposition relative to distance from the coast were more consistent than distance from storm track, and nearly identical for the two deltaic plain events, Katrina and Gustav, as a percent of their total deposition (Fig. 3C). The inland distribution from Hurricane Rita was similar, but the decrease along an inland trend, as a percent of total, occurred more quickly (Fig. 3C). Hurricanes Katrina and Rita both resulted in the deposition of 27 MMT within the first 10 km inland, which was the highest quantity observed in any 10 km interval for the 4 events in this study (Fig. 3D). The total sedimentation from Gustav within this same interval was nearly an order of magnitude lower than Hurricanes Katrina and Rita. At least 80% of the total sedimentation occurred within the first 30 km inland.

### Sediment characteristics

The sediment characteristics in wetlands near Bayou Bienvenue, the area bounded by the Mississippi River on the west and the Mississippi River Gulf Outlet on the east, were similar to that of the surrounding areas following Hurricane Katrina. The maximum observed mineral accretion in this area was 20.81 g cm^−2^, 28 km from the storm path and 5 km from Lake Borgne, while the maximum for the remainder of the study area was 9.92 g cm^−2^, 8 km from the path and 3 km from Breton Sound. The percent mineral sediment in this area was as much as 98%, which was much greater than the 66% observed in wetlands 10 km south of this area. This high mineral content was more similar to that of areas 0 to 15 km from the storm path (µ = 93%). In contrast to Hurricane Katrina, the maximum sedimentation in the other storm events occurred nearest coastal bays and the Gulf of Mexico, and decreased inland.

**Figure 4:**
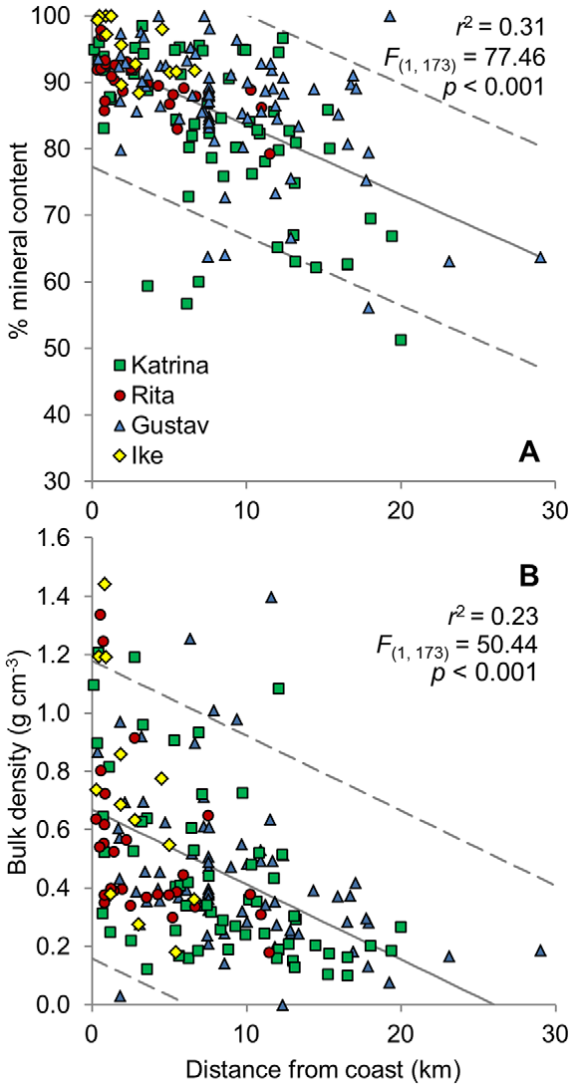
Changing sediment characteristics with distance from coast. Percent mineral content (A) and bulk density (B) with distance from coastline for sediment deposited from Hurricanes Katrina, Rita, Gustav, and Ike. Note that the Y-axis for Figure A begins at 30%. Dashed lines show 95% prediction intervals.

There were significant trends in organic content and bulk density with distance from the coast, but the relationships were noisy (Fig. 4). There was a significant decreasing inland trend of mineral content for all four hurricanes, with the Chenier Plain events being more tightly clustered towards higher mineral content and shorter distances. The sediment bulk density was more variable than mineral content, but decreased along a coastline-to-inland gradient for all events studied. The mean inorganic content within the first 5 km from coast was 92.0%±0.8 SE (86.2%±0.8 for all sites) and the mean bulk density within the same distance was 0.63 g cm^−3^ ±0.04 SE (0.48 g cm^−3^ ±0.02 for all sites).

These results demonstrated that sediment characteristics with respect to distance from the storm track were more variable between depositional events. For this reason, each event was analyzed separately. With increasing distance from track, sediments deposited following Hurricane Rita decreased in mineral content (*r* ^2^ = 0.35, *F*_(1, 26)_ = 13.68, *p* = 0.001) and bulk density (*r* ^2^ = 0.18, *F*_(1, 26)_ = 5.86, *p* = 0.023). The peak bulk density was observed 31 km east of the storm path, which is also where the greatest deposition occurred. The bulk density of Hurricane Katrina sediment samples decreased until 100 km from the hurricane track, and then there was a sharp increase, with the maximum bulk density observed at 148 km from the track. The mineral content of samples collected immediately after Hurricane Gustav decreased slightly with increasing distance from the hurricane track (*r* ^2^ = 0.06, *F*_(1, 72)_ = 4.44, *p* = 0.039), but there was no significant relationship between bulk density and distance from the hurricane track, which initially increased with distance, but then decreased in samples closer to Mississippi River levees. The peak bulk density from Hurricane Gustav was observed 35 km to the east of the hurricane track. Hurricane Ike sediments exhibited similar patterns to the other Chenier Plain event, Rita. Sediment mineral content (*r* ^2^ = 0.49, *F*_(1, 11)_ = 10.74, *p* = 0.007) and bulk density (*r* ^2^ = 0.42, *F*_(1, 11)_ = 7.89, *p* = 0.017) both decreased significantly with distance from the storm track, but there were fewer samples than the other events, and no samples closer than 75 km from the storm track.

**Figure 5:**
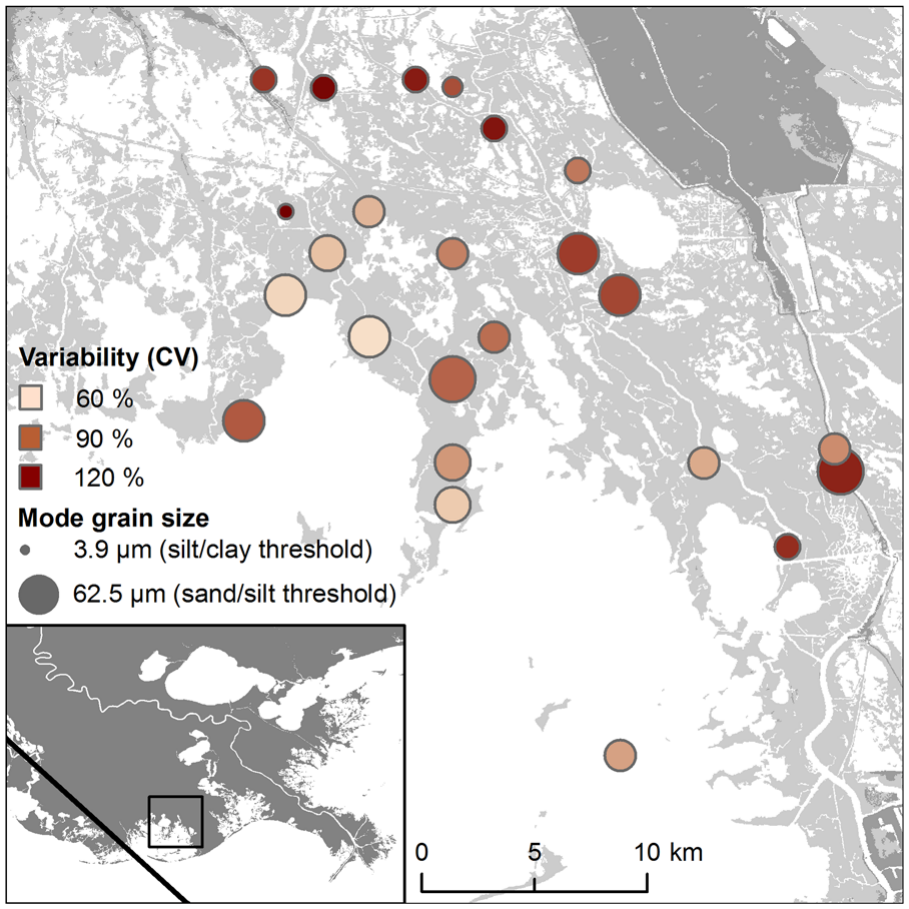
Mode grain size and variability for a subsample of sediments from Hurricane Gustav. The grain sizes decrease (symbol size) and are more poorly sorted (symbol color) with distance from open water. Variability is calculated as the coefficient of variation (CV). Inset shows sample area and track of Hurricane Gustav.

The mode grain size within the subsampled area of Hurricane Gustav decreased with increasing distance from shoreline (*r* ^2^ = 0.25, F_(1, 22)_ = 7.19, *p* = 0.013; Fig. 5). Sediment was more poorly sorted further inland along the same gradient, as determined by increasing coefficients of variation (*r* ^2^ = 0.26, *F*_(1, 22)_ = 7.88, *p* = 0.010). Overall, the mean grain size was 34.8 µm, which is classified as silt. However, 17.3% of the total sample volume was comprised of grains larger than very fine sand (63.4 µm), and the range extended to fine sand (256.9 µm).

## Discussion

The quantity of sediment left on the marsh surface following tropical cyclone events can exceed 10^8^ t yr^−1^ (100 MMT yr^−1^), but the spatial and temporal distribution of these events can vary widely. The amount of sediment deposited on coastal wetlands ranged considerably between the hurricanes, with Hurricane Katrina depositing nearly three times the total sediment of Hurricane Gustav. Hurricane Rita deposited about 70% of the amount left by Hurricane Katrina. Our combined estimate of 116 MMT for Hurricanes Katrina and Rita is 89% of the 131 MMT reported by Turner and others using the same dataset [5], but different analysis methods. This equates to 80% of the modern annual sediment discharge from the Mississippi River being deposited on wetlands alone [24]. Subaqueous deposition, if prorated for inshore waters, increases the total deposition to as high as 135, 67, and 38 MMT for Hurricanes Katrina, Rita, and Gustav, respectively.

A previously published estimate of the total deposition from Hurricane Ike is 16.2 MMT, but the sampling did not include the entire area impacted by the storm [21]. Our estimate of 32.8 MMT in Louisiana for Hurricane Ike reflects differences in sampling methodology and areal coverage, because our sampling design included thinner deposits observed farther inland. The portion of our estimate beyond that sampled in the previous study is 26.8 MMT. We estimate the combined total from both studies at 43 MMT, making Hurricane Ike most similar to Hurricane Rita in terms of total deposition, although this estimate has a larger margin of uncertainty because it combines two sampling and analysis methods.

Sediment deposition tended to be greatest just east of the storm path, and nearer the coastline, for the two storms making landfall west of the Mississippi River. The areas of high deposition generally coincided with the highest storm surges in previously reported models, but modeled surges covered a broader area [3], [25]. The distribution of sediment in Louisiana wetlands following Hurricane Katrina was mostly west of the path, due to the strong easterly winds that preceded the storm and drove the storm surge into the east-oriented estuary. This effect is demonstrated by previous storm surge events [26] and simulated events [3].

The majority of sedimentation (80%±5 SE) occurred within the first 20 km inland, and declined exponentially. With distance from the storm track, about half (48%±10 SE) of the total occurred within the first 50 km, but the distribution was more variable. Comparing between the three events, there are remarkably consistent depositional patterns along a shoreline-to-inland gradient. These patterns were more variable with distance from the path, because the high sedimentation during Hurricane Katrina was closest to its storm track, whereas the other two events peaked 50 km east of the storm tracks. A similar relationship with distance from landfall was reported for Hurricane Ike, where deposition was greatest about 40 km east of landfall, and then decreased with distance [21]. The most distant sedimentation averaged 157 km from the storm track (Table 1), which compares well to the 130 km maximum observed after Hurricane Andrew [27]. The Chenier Plain events spread sediment farther along the coast than the Deltaic Plain landfalls, but this could also result from characteristics of the storms rather than landfall location.

The high deposition we observed in the Bayou Bienvenue area following Hurricane Katrina was anomalous compared to nearby sample locations and the otherwise decreasing inland trend, but is consistent with high storm surge energy reported for that area [28], [29]. The spatial distribution of wetland sedimentation for Hurricane Rita, which was up to 8.7 cm thick 0.3 km from the coast and diminishing to 0.1 cm 12 km inland, is consistent with the reported ranges of 7.6 to 188 cm on and directly behind ridges that led to increased over-wash [30]. This wetland sedimentation from Hurricane Rita was described as two distinct sequences: an underlying thin layer comprised of fine sand and mud overlain by a coarser, thicker layer comprised mainly of sand, but with a more limited areal extent [31]. The finer sediment layer was rich in foraminiferal remains, suggesting a more offshore origin, whereas the higher sand content of the coarser layer resembled beach and dune overwash.

The reduction in storm surge as it propagates shoreward is reflected in observations of deposition ranging from offshore to inshore ponds and wetlands. Offshore deposition in 2005 was at least 10 times greater than our estimate for wetlands [9]. This offshore deposition may remain as in-tact event horizons for decades [12]. Inshore deposition following hurricanes can also be significant [13]–[15], but the spatial extent and how well it compares to deposition in surrounding wetlands remains unclear. Although these estimates were within range of observations of wetland deposition for Hurricanes Andrew and also Rita/Katrina, local bathymetry and channel morphology can play a major role in determining whether erosion or deposition will occur [32].

Landscape-scale wetland sedimentation events have been observed in other areas following tropical cyclones, such as the Florida coast. In southwest Florida following Hurricane Andrew in 1992, an average deposition of 7 cm (range: 0–20 cm) across an area of 110 km^2^ was observed [13]. The mean organic content of these sediments was 8% (range: 4–22%). We observed a greater organic content in Louisiana of 14% (range: 2–49%) for all three hurricanes, which is consistent with the contrast between bottom sediments in coastal Louisiana and Florida Bay.

The areas of highest mineral content and bulk density were near the coast, which is where storm surge energies were greatest, and decreased inland. Similar trends in organic content and bulk density were observed following Hurricane Andrew in Louisiana [18]. In this same location studied during Hurricane Andrew, we observed anomalously high bulk density and mineral content that strayed from the overall distance-from-track trends following the 2005 hurricanes. This area was 148 km from the nearest storm path, but near Atchafalaya Bay, and may have been the result of overlapping influences of both 2005 hurricanes as well as the nearby Atchafalaya River acting as a sand source.

Although grain size measurements were more spatially limited, an inland trend of decreasing grain size was also observed in Florida following Hurricane Andrew, but the Florida sediments were predominantly carbonate-rich sand [13]. There was a sharper decline in sediment grain size for Hurricane Andrew in Florida than we observed for Gustav in Louisiana, which was attributed to the relatively short storm surge duration resulting from Andrew's small eye and rapid speed. The overall decreasing inland trends in bulk density, mineral content, and grain size are indicative of storm energy attenuation by marshes and shallow inshore waters, whereby the heavier materials are dropped from suspension closer to the Gulf. Some variability in bulk density, however, may reflect consolidation that occurred following their initial deposition, because sampling did not occur for several months for some samples. The residual variability in organic content, bulk density, and grain size, despite being statistically significant with distance from the coast, likely results from smaller scale factors that also affect sediment distribution such as vegetation and local geomorphology. Sediment deposition may also vary on a more local scale because of variations in local bathymetry or channel morphology [32], [33], or on an even smaller scale such as differing vegetation types or stem densities [17], [34]. From a coast-wide perspective, however, these effects appear to be overshadowed by variations in storm energy.

Some of the sediment deposited during storms from inshore sources may include newly eroded wetland soils. The amounts of this lateral erosion of coastal wetlands during tropical cyclones can vary widely; some effects recover within months, while others may remain for decades [35]. Following Hurricanes Katrina and Rita in 2005, 525 km^2^ of new water area was observed, but a portion of this may be due to the removal of floating aquatic vegetation rather than wetland [35]. Many areas lost whole blocks of marsh peat, forming linear scars parallel to the wind direction, accordion-like features, and rolled up vegetation mats. Although large areas of open water appeared after the 2005 and 2008 hurricane seasons, some recovery occurred within a couple years, but the long-term trend of land loss continued in many areas [36]. Much of the land loss from the 2005 hurricane season occurred in areas where the wetland substrate had been weakened due to the introduction of Mississippi River water, which led to changes in vegetation composition [37] as well as increased soil decomposition and decreased root biomass [38].

The contribution of these powerful, but infrequent, events to the long-term sedimentary record of wetlands may vary widely [39], and be significant [40]. McKee and Cherry [41] examined what happened in wetlands that had sediments deposited during the 2005 hurricane season. They observed that, compared to a control site receiving less sediment, that the wetlands under the strong influence of the hurricanes had a lower soil elevation loss. Sediment deposited in wetlands of the Chenier Plain from Hurricane Rita in 2005 had been incorporated into the soil profile when re-examined in 2007, and the authors concluded that these types of events contributed one to two thirds of the sediment in the soil profile [42]. In a study covering a longer time scale, Turner and others [40] reported peaks in mineral content of salt marsh soils as early as 1880 that coincided with periods of increased hurricane frequency. A similar study also noted peaks in sand content of wetland soils in New England that were correlated with known storm events [43]. Peaks in sand content beyond the historical record were observed as early as 1450, indicating that, although storm-deposited sediment may become reworked by physical or biological processes, some of this sediment is incorporated into the long term sediment record. Variations in inorganic sedimentation rates in a North Sea salt marsh were found to be driven by storm frequency and intensity, with periods of intense storm tides corresponding to high sedimentation for the 70-year period studied [44].

Tropical cyclone events represent both potential agents of land loss, but also a significant input of sediment to the wetland soil profile. In a deltaic system with rates of isostatic sea level rise approaching one cm yr^−1^ [45], storm-driven inputs of sediment from nearshore or offshore may be an important, if not dominant, component of coastal wetland inorganic accretion [40]. The Mississippi River is the ultimate source of most of this sediment, via the continental shelf, but its transport from offshore to inshore during infrequent, but intense, events represents an important component of coastal sedimentation, and may represent the greatest allogenic source of sediment for coastal wetlands in abandoned delta lobes. Although we have described massive sedimentation following these four events, further research is needed to identify how these types of events contribute to the long-term sediment budget for coastal Louisiana wetlands. A more complete understanding of the factors driving sedimentation events for coastal wetlands will lead to the implementation of more efficient and effective coastal restoration actions.
